# Move more, scroll less? Physical activity, smartphone use, and subjective body shape perception in adolescents: within-person associations in gender-stratified analyses

**DOI:** 10.3389/fpubh.2026.1927639

**Published:** 2026-09-22

**Authors:** Juhyeong Kim, Sangwook Kang

**Affiliations:** Department of Physical Education, College of Education, Seoul National University, Seoul, Republic of Korea

**Keywords:** adolescent body image, fixed-effects models, gender-stratified analysis, physical activity promotion, screen time

## Abstract

**Introduction:**

Subjective body shape perception is an important adolescent health issue linked to psychological well-being and weight related behaviors. This study examined within-person associations among subjective body shape perception, physical activity, and smartphone use separately among Korean adolescent boys and girls.

**Methods:**

Using five-wave data from the Korean Youth Health Behavior Panel Survey (2019–2023), we estimated two-way fixed-effects models controlling for time-invariant individual characteristics and wave-specific factors. The analytic sample included 4,518 adolescents (boys: 2,341; girls: 2,177), contributing 18,721 observations.

**Results:**

Among boys, within-person increases in physical activity were associated with lower subjective body shape perception scores (B = −0.0074, *p* < 0.05), whereas weekday and weekend smartphone use were not significantly associated with subjective body shape perception. Among girls, increases in weekday smartphone use were associated with higher subjective body shape perception scores (B = 0.0129, *p* < 0.05), while physical activity was not associated with subjective body shape perception. In additional models, physical activity was associated with lower smartphone use scores among girls on both weekdays (B = −0.0422, *p* < 0.001) and weekends (B = −0.0294, *p* < 0.01).

**Discussion:**

These findings suggest that physical activity, smartphone use, and subjective body shape perception may be connected in behavioral patterns observed separately among boys and girls. Future research and intervention studies may consider gender-sensitive approaches that integrate physical activity promotion, smartphone use awareness, and media literacy to support healthier body-related perceptions during adolescence.

## 1 Introduction

Body image represents a key determinant of adolescent health and psychological well-being. Body image is a multidimensional construct that encompasses how individuals perceive, think about, feel about, and behave toward their bodies (1). Within this multidimensional construct, the present study focuses specifically on subjective body shape perception, or adolescents’ self-perceived body shape along a thinness–fatness continuum. Therefore, this construct should be interpreted as one perceptual component of body image rather than as overall body image. It exists on a continuum ranging from accurate and generally positive perceptions of one’s body to inaccurate and negative, unhealthy perceptions (2, 3). Prior research has consistently shown that negative body image is associated with obesity and insufficient physical activity (4). In addition, evidence that body dissatisfaction is linked to psychological problems such as low self-esteem, depression and anxiety, and eating disorders underscores that body image plays a critical role in health and public well-being (5–7).

In particular, adolescence is a period in which body image is rapidly formed and reshaped as rapid physical growth, the heightened importance of peer relationships, and increased exposure to media intersect with physical, social, cultural, and psychological changes (8–10). As a key factor in adolescents’ social relationship formation, body image influences health and well-being (11), and distorted body image may lead to inappropriate weight-control behaviors that threaten adolescents’ health (12). Moreover, body image is understood not as a fixed personal trait but as a developmental construct that can change across the life course (13). Because adolescence involves multiple developmental transitions occurring simultaneously, body image may be particularly sensitive to fluctuation during this period. Body size and body shape misperception may involve not only perceived fatness but also perceived muscularity or body shape (14), and these dimensions may become particularly salient during adolescence when the body is undergoing rapid developmental change. Therefore, it is necessary to identify in detail the everyday experiences and behavioral factors that help explain changes in body image at this stage, and one key factor warranting attention is physical activity.

Physical activity is a modifiable health behavior that may shape adolescents’ body-related perceptions through both physiological and psychological pathways. Previous reviews and meta-analyses suggest that exercise and physical activity are generally associated with more positive body image outcomes (15–18). Physiologically, physical activity may be related to body-related perceptions through changes in weight status, body composition, physical fitness, and functional capacity. Psychologically, physical activity may shape body-related perceptions by providing repeated experiences of using, regulating, and testing the body in everyday contexts, including bodily competence, mastery, self-efficacy, and goal attainment (19, 20). From this perspective, repeated experiences of performance, goal attainment, and bodily competence may help adolescents view their bodies less negatively. Thus, physical activity may function not only as a health behavior, but also as a behavioral context associated with healthier body-related self-perceptions.

At the same time, adolescents’ body-related perceptions may be shaped not only by embodied experiences such as physical activity, but also by digital experiences mediated through smartphones. Smartphone use, particularly when it involves social networking environments, may increase exposure to idealized appearance-related content and facilitate upward social comparison (21–23). From the perspective of social comparison theory, adolescents may evaluate their own bodies by comparing themselves with peers, celebrities, or influencers when objective standards for appearance are unclear or socially contested (24). Because adolescence is a developmental period in which peer attention and social approval are highly salient, repeated exposure to curated images of peers, celebrities, or influencers may encourage adolescents to evaluate their own appearance against idealized standards (25–27). In this sense, greater smartphone use may reflect more frequent immersion in appearance-comparison contexts, which may in turn be associated with changes in subjective body shape perception. Within this broader domain of body image, the present study focuses specifically on subjective body shape perception—how adolescents perceive their own body shape—because this evaluative dimension may be especially relevant to everyday self-assessment and weight-related behavior.

Although adolescents’ body-related perceptions may also be shaped by factors such as social media-specific activities, bullying, sleep, dietary behaviors, and mental health, the present study focused on physical activity and smartphone use because they are common and modifiable daily behaviors. These two behaviors capture distinct but potentially connected contexts of adolescent self-evaluation: physical activity reflects embodied experiences of competence, function, and physical change (19, 20), whereas smartphone use reflects digital environments in which appearance-related comparison may occur. Examining these behaviors together may therefore provide a focused but relevant framework for understanding behavioral contexts linked to subjective body shape perception.

Physical activity and smartphone use may also be behaviorally interconnected. Physical activity may restructure adolescents’ daily routines and time allocation, potentially displacing screen-based activities. Prior studies have reported that higher levels of physical activity are associated with lower smartphone overuse or dependency and have suggested that physical activity may provide alternative sources of reward that reduce reliance on smartphone use (28–30). Accordingly, adolescents who become more physically active may spend less time on smartphones, whereas decreases in physical activity may coincide with more screen-based routines. Examining physical activity and smartphone use together may therefore provide a more integrated understanding of the behavioral context in which body shape perception changes, particularly by clarifying whether changes in physical activity co-occur with changes in smartphone use.

These relationships may differ by gender. Girls are more consistently exposed to thin-ideal appearance norms and generally report higher levels of negative body-related outcomes such as body dissatisfaction, whereas boys’ body evaluations are shaped not only by appearance-based standards but also by functional and physique-related criteria (31–34). Recent evidence suggests that clinically significant body dissatisfaction is observed in both adolescent boys and girls, although its prevalence and psychological correlates may differ by gender (35). In addition, appearance-focused media environments and social comparison pressures have been shown to operate more strongly among girls (36, 37). For girls, subjective body shape perception may therefore be particularly sensitive to perceived fatness, thin-ideal internalization, and appearance-based social comparison. For boys, however, body-related self-evaluation may also involve perceived muscularity, physical function, and bodily competence (17). Therefore, lower scores on a thinness–fatness perception scale should not automatically be interpreted as healthier body image in all cases. Prior research also suggests that adolescents’ body image perception differs according to sex, weight status, and sports practice, further supporting the need to examine these associations separately among boys and girls (38). Accordingly, gender-stratified analysis may help clarify whether the associations among physical activity, smartphone use, and subjective body shape perception show different patterns among boys and girls.

Despite these theoretical and empirical links, much of the existing literature has relied on cross-sectional designs (39–41). Such designs make it difficult to determine whether observed associations reflect within-person behavioral change or stable between-person differences. Relatively stable characteristics—such as appearance sensitivity, social comparison tendencies, or family environment—may simultaneously influence physical activity, smartphone use, and body-related perceptions. To address this limitation, the present study uses 5 waves of panel data and fixed-effects models to examine whether within-person changes in physical activity and smartphone use are associated with changes in adolescents’ subjective body shape perception. We further stratify analyses by gender and examine whether physical activity is associated with smartphone use, with the goal of clarifying behavioral contexts linked to body shape perception in analyses conducted separately among boys and girls.

Specifically, this study addressed two research questions: first, whether within-person changes in physical activity and smartphone use are associated with changes in subjective body shape perception among boys and girls, respectively; and second, whether within-person changes in physical activity co-occur with changes in smartphone use among boys and girls.

## 2 Methods

### 2.1 Data collection and study participants

This study used data from the Korean Youth Health Behavior Panel Survey (KYHPS), a longitudinal panel survey established and administered by the Korea Disease Control and Prevention Agency to investigate determinants and developmental patterns of adolescents’ health behaviors (42). The target population of the KYHPS was sixth grade students enrolled in elementary schools in Korea in 2019. The sampling frame was based on the national list of fifth grade students as of April 2018, and participants were followed annually. The present study used the first five waves collected between 2019 and 2023. At baseline, participants were sixth-grade students, who are typically 11–12 years old in the Korean school system.

The KYHPS used a multistage stratified cluster sampling design, with the target population defined as sixth grade students nationwide in 2019. In the stratification stage, 25 strata were constructed by considering province/metropolitan city and regional size. Schools were selected as the primary sampling units, and classes within selected schools were selected as secondary sampling units. The analytic dataset provides survey design variables, including strata, sampling clusters, and longitudinal and cross-sectional weights. The baseline wave included 5,051 participants (2,634 boys and 2,417 girls), and the present study combined data from 5 waves collected between 2019 and 2023.

Because the analysis relied on individual fixed-effects models, the analytic sample was restricted to adolescents observed at least twice across the 5 waves. We further applied a complete case approach, retaining only observations with no missing values for the dependent variables, focal independent variables, and covariates included in each model. Under these criteria, the final analytic sample included 4,518 adolescents (2,341 boys and 2,177 girls), contributing 18,721 observations (9,523 for boys and 9,198 for girls).

### 2.2 Data analysis

To examine how within-person changes in adolescents’ physical activity and smartphone use are associated with changes in subjective body shape perception, this study applied fixed-effects panel regression models to five-wave longitudinal data. A key challenge in observational research is to distinguish whether observed associations reflect changes in time-varying behaviors (e.g., physical activity and smartphone use) or are confounded by individuals’ relatively stable characteristics. Unlike experimental studies, observational designs are vulnerable to endogeneity because unmeasured or time-invariant individual traits may affect both the independent and dependent variables (43), potentially leading to over or underestimation of effects.

Fixed-effects panel regression addresses this concern by absorbing time-invariant individual factors into individual fixed effects, thereby enabling estimation based on within-person variation across repeated observations (44, 45). In addition to individual fixed effects, we included wave fixed effects to account for period-specific influences shared by all participants (e.g., social or environmental changes). Analyses were conducted using complete case observations for variables required in each model.

Models were specified as follows. First, subjective body shape perception was modeled as the dependent variable, estimating (1) a baseline model including only control variables (Model 1) and (2) an extended model adding physical activity and weekday/weekend smartphone use (Model 2). Second, to test whether physical activity was associated with smartphone use, weekday and weekend smartphone use were separately specified as dependent variables, and Model 1 (controls only) was compared with Model 2 (controls plus physical activity) using the same analytic sample. All analyses were stratified by gender to align with the study questions and to describe within-person associations separately among boys and girls. All models included individual and wave fixed effects, and standard errors were clustered at the individual level to account for within-person correlation in repeated measures. Because the main analytic aim was to estimate within-person associations over time, individual-level clustering was used to address the no independence of repeated observations from the same adolescent. Although the KYHPS provides survey design variables for strata, sampling clusters, and weights, the present fixed-effects analyses did not additionally incorporate the original sampling cluster structure. Analyses were performed in R version 4.4.1.

### 2.3 Measurements

All study variables were drawn from the KYHPS questionnaire. Unless otherwise noted, higher scores indicate higher levels of the corresponding construct.

#### 2.3.1 Physical activity

To assess adolescents’ physical activity, we used the KYHPS item on frequency of moderate to vigorous physical activity (MVPA). Participants were asked: “During the past 7 days, on how many days did you engage in physical activity for a total of at least 60 minutes per day that made your heart beat faster than usual or made you feel out of breath?” Responses were recorded on an 8 point scale ranging from 1 (none in the past 7 days) to 8 (7 days/week), with higher scores indicating more frequent engagement in MVPA.

#### 2.3.2 Smartphone use time

Adolescents’ smartphone use time was measured separately for weekdays and weekends. Participants were asked: “During the past 7 days, how many hours per day did you use a smartphone on average?” and responded separately for weekdays and weekends. Responses were recorded on a 9 point scale (1 = not at all, 2 = less than 30 min, 3 = 30 min to <1 h, 4 = 1 to <2 h, 5 = 2 to <3 h, 6 = 3 to <4 h, 7 = 4 to <5 h, 8 = 5 to <6 h, 9 = 6 h or more), with higher scores indicating longer average daily smartphone use. This measure captured total duration of smartphone use and did not distinguish between specific purposes, activities, or content types.

#### 2.3.3 Subjective body shape perception

Subjective body shape perception was assessed with a single item: “How do you think about your body shape?” Responses ranged from 1 (very thin) to 5 (very overweight), with higher scores indicating perceiving oneself as heavier. This item was interpreted as a single item measure of perceived body shape along a thinness–fatness continuum, rather than as a comprehensive measure of overall body image Table 1.

**Table 1:** Number of observations by wave.

	Wave 1	Wave 2	Wave 3	Wave 4	Wave 5
Boys	2,063	1,784	1,834	1,952	1,890
Girls	1,998	1,777	1,831	1,835	1,757

#### 2.3.4 Control variables

We included BMI as an indicator of actual body size that may be directly related to subjective body shape perception (46). BMI was calculated as weight in kilograms divided by height in meters squared (kg/m^2^), using the height and weight information provided in the KYHPS. Perceived household economic status was included to account for socioeconomic differences that may shape health behaviors, media use, and body-related attitudes (47). Higher scores for perceived household economic status indicated higher perceived economic status.

We also controlled parental restriction of smartphone use and whether smartphone use was permitted during meals, both of which may reflect family rules structuring adolescents’ smartphone use. For parental restriction of smartphone use, higher scores indicated stricter restriction of adolescents’ daily smartphone use. For smartphone use restriction during meals, higher scores indicated stricter restriction during mealtimes. Finally, participation in school physical education classes was included because it represents institutionally provided opportunities for physical activity and may also be related to body-related perceptions (48). Higher scores for participation in school physical education classes indicated greater participation.

## 3 Results

Wave-specific descriptive statistics (means and standard deviations) for the key study variables are presented in Table 2, stratified by gender. Physical activity scores generally declined over waves for both boys and girls, and boys consistently reported higher levels of physical activity than girls across all waves. Weekday and weekend smartphone use increased over time in both groups, with girls reporting higher levels of smartphone use than boys. Mean subjective body shape perception scores changed in different directions across waves among boys and girls: scores tended to decrease among boys, whereas they tended to increase among girls.

**Table 2:** Descriptive statistics of key variables by gender and wave.

Gender	Variable	Wave 1	Wave 2	Wave 3	Wave 4	Wave 5
Boys	Physical activity	4.69 (2.38)	4.02 (2.24)	3.91 (2.16)	4.39 (2.15)	3.91 (2.01)
	Weekday smartphone use time	4.70 (1.73)	4.80 (1.67)	5.25 (1.64)	5.31 (1.51)	5.28 (1.61)
	Weekend smartphone use time	5.88 (1.95)	5.63 (1.77)	6.24 (1.70)	6.40 (1.66)	6.48 (1.63)
	Subjective body shape perception	3.09 (0.97)	3.15 (1.04)	3.07 (1.03)	2.99 (1.06)	2.95 (1.01)
	BMI	20.38 (3.40)	21.40 (3.61)	21.82 (3.65)	21.84 (3.63)	22.07 (3.40)
	Household economic status	3.49 (0.80)	3.42 (0.78)	3.33 (0.74)	3.32 (0.73)	3.31 (0.73)
	Daily smartphone use restriction	2.97 (0.89)	2.80 (0.88)	2.51 (0.90)	2.35 (0.92)	2.08 (0.87)
	Smartphone use restriction during meals	3.02 (0.89)	2.95 (0.89)	2.82 (0.86)	2.70 (0.87)	2.60 (0.86)
	Level of participation in physical education classes	3.68 (0.55)	3.61 (0.59)	3.55 (0.64)	3.53 (0.67)	3.57 (0.64)
	Observation (*n*)	2,063	1,784	1,834	1,952	1,890
Girls	Physical activity	3.65 (2.02)	3.16 (1.91)	3.14 (1.96)	3.29 (1.87)	2.80 (1.67)
	Weekday smartphone use time	5.26 (1.78)	5.51 (1.81)	5.86 (1.71)	5.85 (1.59)	5.74 (1.70)
	Weekend smartphone use time	6.34 (2.07)	6.42 (1.90)	6.97 (1.70)	7.16 (1.59)	7.09 (1.54)
	Subjective body shape perception	3.17 (0.88)	3.23 (0.88)	3.28 (0.87)	3.29 (0.89)	3.30 (0.86)
	BMI	19.39 (2.89)	19.92 (2.89)	20.35 (2.93)	20.56 (2.92)	20.82 (2.85)
	Household economic status	3.38 (0.77)	3.31 (0.74)	3.25 (0.72)	3.20 (0.73)	3.21 (0.72)
	Daily smartphone use restriction	2.83 (0.90)	2.69 (0.88)	2.38 (0.86)	2.20 (0.86)	2.03 (0.83)
	Smartphone use restriction during meals	3.04 (0.87)	2.97 (0.87)	2.73 (0.88)	2.62 (0.86)	2.52 (0.86)
	Level of participation in physical education classes	3.41 (0.67)	3.38 (0.67)	3.31 (0.73)	3.28 (0.77)	3.35 (0.72)
	Observation (n)	1,998	1,777	1,831	1,835	1,757

Values are presented as mean (standard deviation). Physical activity was measured on an 8-point frequency scale from 1 (none in the past 7 days) to 8 (7 days/week). Smartphone use was measured on a 9-point average daily use-time scale from 1 (not at all) to 9 (6 h or more). Subjective body shape perception was measured on a 5-point scale from 1 (very thin) to 5 (very overweight).

### 3.1 Association between changes in physical activity and smartphone use and changes in perceived body shape

Table 3 presents gender-stratified fixed-effects regression results examining the associations of physical activity and smartphone use with subjective body shape perception. Model 1 included only the control variables, whereas Model 2 added physical activity and weekday and weekend smartphone use. Model 2 therefore represents the main result for examining whether within-person changes in physical activity and smartphone use were associated with changes in subjective body shape perception.

**Table 3:** Fixed-effects regression results for subjective body shape perception: physical activity and smartphone use time.

Variable	Model 1		Model 2	
	B [95% CI]	*p*	B [95% CI]	*p*
Boys				
BMI	**0.1606** **[0.1510, 0.1702]**	**<0.001**	**0.1602** **[0.1506, 0.1699]**	**<0.001**
Household economic status	−0.0182 [−0.0421, 0.0056]	0.135	−0.0174 [−0.0413, 0.0064]	0.153
Daily smartphone use restriction	−0.0024 [−0.0197, 0.0149]	0.786	−0.0024 [−0.0197, 0.0149]	0.783
Smartphone use restriction during meals	0.0145 [−0.0038, 0.0328]	0.120	0.0147 [−0.0035, 0.0330]	0.114
Level of participation in physical education classes	−0.0159 [−0.0423, 0.0105]	0.237	−0.0132 [−0.0399, 0.0134]	0.330
Physical activity			**−0.0074** **[−0.0140, −0.0009]**	**<0.05**
Weekday smartphone use time			−0.0039 [−0.0154, 0.0075]	0.499
Weekend smartphone use time			0.0027 [−0.0086, 0.0139]	0.645
Girls				
BMI	**0.1499** **[0.1386, 0.1612]**	**<0.001**	**0.1492** **[0.1379, 0.1605]**	**<0.001**
Household economic status	**−0.0269** **[−0.0500, −0.0039]**	**<0.05**	**−0.0267** **[−0.0498, −0.0036]**	**<0.05**
Daily smartphone use restriction	0.0116 [−0.0047, 0.0279]	0.162	0.0127 [−0.0036, 0.0291]	0.128
Smartphone use restriction during meals	0.0054 [−0.0117, 0.0226]	0.536	0.0067 [−0.0105, 0.0238]	0.446
Level of participation in physical education classes	**−0.0206** **[−0.0410, −0.0002]**	**<0.05**	−0.0186 [−0.0390, 0.0019]	0.076
Physical activity			−0.0030 [−0.0097, 0.0038]	0.389
Weekday smartphone use time			**0.0129** **[0.0023, 0.0235]**	**<0.05**
Weekend smartphone use time			−0.0022 [−0.0138, 0.0094]	0.713

B values are unstandardized coefficients from fixed-effects regression models. Significant values are in bold.

Among boys, BMI was positively associated with subjective body shape perception in both models. In Model 2, higher levels of physical activity were associated with lower subjective body shape perception scores (*B* = −0.0074, *p* < 0.05), indicating that within the same individual, increases in physical activity coincided with lower scores on the thinness–fatness perception scale. In contrast, weekday and weekend smartphone use were not significantly associated with perceived body shape among boys.

Among girls, BMI was positively associated with subjective body shape perception in both models, and household economic status was negatively associated with subjective body shape perception. In Model 2, increases in weekday smartphone use were significantly associated with higher subjective body shape perception scores (B = 0.0129, *p* < 0.05), suggesting that greater weekday smartphone use coincided with higher scores on the thinness–fatness perception scale. Physical activity and weekend smartphone use were not significantly associated with perceived body shape among girls. Because subjective body shape perception was measured on a 5-point scale, the coefficients should be interpreted as changes in scale scores.

### 3.2 Association between changes in physical activity and changes in smartphone use time

Table 4 presents fixed-effects estimates of the association between physical activity and smartphone use time, stratified by gender and day type (weekday vs. weekend). In these models, weekday and weekend smartphone use time were specified as dependent variables. The models included the same control variables as Table 3, with physical activity added as the independent variable.

**Table 4:** Fixed-effects regression results for smartphone use time: associations with physical activity.

Variable	Weekday smartphone use time		Weekend smartphone use time	
	B [95% CI]	*p*	B [95% CI]	*p*
Boys				
BMI	−0.0038 [−0.0275, 0.0200]	0.756	0.0136 [−0.0119, 0.0392]	0.296
Household economic status	−0.0075 [−0.0729, 0.0580]	0.823	−0.0260 [−0.0930, 0.0409]	0.446
Daily smartphone use restriction	**−0.1051** **[−0.1557, −0.0544]**	**<0.001**	**−0.1127** **[−0.1639, −0.0614]**	**<0.001**
Smartphone use restriction during meals	−0.0265 [−0.0772, 0.0242]	0.306	−0.0484 [−0.1020, 0.0052]	0.077
Level of participation in physical education classes	**−0.0992** **[−0.1688, −0.0296]**	**<0.01**	**−0.0689** **[−0.1359, −0.0020]**	**<0.05**
Physical activity	−0.0155 [−0.0331, 0.0020]	0.083	−0.0016 [−0.0201, 0.0170]	0.869
Girls				
BMI	**0.0616** **[0.0315, 0.0916]**	**<0.001**	**0.0480** **[0.0180, 0.0781]**	**<0.01**
Household economic status	0.0037 [−0.0658, 0.0732]	0.916	−0.0317 [−0.0995, 0.0362]	0.361
Daily smartphone use restriction	**−0.0963** **[−0.1463, −0.0462]**	**<0.001**	**−0.1356** **[−0.1872, −0.0841]**	**<0.001**
Smartphone use restriction during meals	**−0.0937** **[−0.1452, −0.0423]**	**<0.001**	**−0.1211** **[−0.1737, −0.0685]**	**<0.001**
Level of participation in physical education classes	**−0.1033** **[−0.1663, −0.0402]**	**<0.01**	**−0.0996** **[−0.1611, −0.0382]**	**<0.01**
Physical activity	**−0.0422** **[−0.0629, −0.0215]**	**<0.001**	**−0.0294** **[−0.0495, −0.0093]**	**<0.01**

B values are unstandardized coefficients from fixed-effects regression models. Significant values are in bold.

Among boys, increases in physical activity were not statistically significantly associated with weekday smartphone use (B = −0.0155, *p* = 0.08) or weekend smartphone use (B = −0.0016, *p* = 0.87). In contrast, among girls, increases in physical activity were significantly associated with lower smartphone use scores both on weekdays (B = −0.0422, *p* < 0.001) and on weekends (B = −0.0294, *p* < 0.01). These findings suggest that, within individuals, periods of higher physical activity co-occurred with lower smartphone use scores, particularly among girls. Because smartphone use was measured on a 9-point categorical use-time scale, the coefficients should be interpreted as changes in smartphone-use category scores rather than exact hours of use.

## 4 Discussion

This study used fixed-effects panel regression models and 5 waves of adolescent panel data to examine whether within-person changes in physical activity and smartphone use were associated with changes in subjective body shape perception, separately for boys and girls. The findings showed different patterns in the models estimated separately for boys and girls. Among boys, increases in physical activity were associated with lower subjective body shape perception scores, whereas smartphone use was not significantly associated with subjective body shape perception. Among girls, increases in weekday smartphone use were associated with higher subjective body shape perception scores, whereas physical activity was not associated with body shape perception. In addition, among girls, increases in physical activity were associated with lower smartphone use on both weekdays and weekends. Taken together, these findings suggest that adolescents’ subjective body shape perception may be embedded in different behavioral contexts depending on gender.

Among boys, the association between increased physical activity and lower body shape perception scores suggests that body-related self-evaluation may be linked to embodied experiences of the body. This interpretation is broadly consistent with prior evidence showing beneficial links between physical activity and body image among males (17, 18, 49, 50). It is also consistent with evidence that sports practice, sex, and weight status are jointly related to adolescents’ body image perception, suggesting that physical activity may be meaningful for body-related perceptions in gender-specific ways (38). Physical activity may be associated with boys’ body-related perceptions not only through visible physical change, but also through repeated experiences of competence and bodily efficacy (16, 19, 20). In this sense, physical activity may provide adolescents with opportunities to experience the body as active, capable, and functional, which may be relevant to how boys evaluate their body shape.

In interpreting these findings, however, it should be noted that the dependent variable in this study is not overall body image but rather subjective body shape perception (i.e., perceived thinness vs. fatness). Male body image is shaped not only by weight-related evaluations but also by male-specific dimensions such as drive for muscularity, which may play a central role in how boys evaluate their bodies (17). Research on body size and body shape misperception also suggests that body-related misperception may involve perceived body size, shape, and muscularity rather than fatness alone (14). Relatedly, Olivardia et al. (51) noted that thinness per se may not constitute a particularly meaningful body image indicator for males. Therefore, lower scores on the subjective body shape perception scale should not automatically be interpreted as healthier overall body image among boys. Taken together, these considerations suggest that the role of physical activity in this study should not be taken as representative of boys’ overall body image; rather, it should be interpreted as an association specific to changes in perceived body shape. Future research should therefore examine the effects of physical activity across a wider range of body image indicators to better capture the multidimensional nature of body image.

Among girls, weekday smartphone use was positively associated with subjective body shape perception, suggesting that greater digital exposure in everyday life may be linked to higher perceived body shape scores. This finding is consistent with prior research showing that internet and social media use may heighten body-related concerns among adolescent girls, particularly when use involves appearance focused content and social comparison (26, 27). Given that peers are a particularly influential reference group for girls (36), weekday smartphone use may be especially salient because it is embedded within school routines and peer contexts, where peer norms and appearance-related comparisons may be more frequent and psychologically meaningful. Repeated exposure to curated and idealized images may encourage upward comparison and, in turn, more negative evaluations of one’s own body shape. This interpretation aligns with Festinger’s social comparison theory (22), which proposes that when objective standards are limited, individuals rely on others as benchmarks for self-evaluation. However, this interpretation should be treated cautiously because the smartphone use measure captured total use time and did not distinguish between specific purposes, activities, or content types. Therefore, the observed association among girls cannot be attributed specifically to appearance-focused social media exposure.

The findings for girls also suggest that physical activity may matter in a different way. Although physical activity was not associated with body shape perception, increases in physical activity were associated with lower smartphone use on both weekdays and weekends. These results may reflect changes in daily routines, time allocation, or activity patterns in which periods of higher physical activity co-occur with lower smartphone use. This pattern is consistent with prior work suggesting that physical activity can restructure daily routines and reduce smartphone dependence by displacing screen-based time or providing alternative sources of reward (28–30). However, these results should not be interpreted as evidence of mediation, because the present study did not formally test whether reduced smartphone use explains the association between physical activity and body shape perception.

The negative association between physical activity and smartphone use among girls may be interpreted in relation to the time-displacement hypothesis, which suggests that activities may compete for adolescents’ limited discretionary time. However, displacement should not be assumed as a deterministic mechanism, because recent longitudinal evidence suggests that mobile phone use does not necessarily displace physical activity, sleep, or other beneficial activities in a simple or uniform way (52). Similarly, the Goldilocks hypothesis suggests that the implications of screen-based activity may depend on the level and context of use rather than reflecting a uniformly harmful exposure (53). Thus, the present findings should be interpreted as within-person co-occurrence between physical activity and smartphone use, rather than evidence that physical activity directly displaces smartphone use.

Next, to better understand the behavioral context in which adolescents’ body shape perception is embedded, this study examined whether changes in physical activity co-occur with changes in smartphone use time. Among girls, increases in physical activity were significantly associated with decreases in smartphone use time on both weekdays and weekends. This pattern is consistent with Linden et al. (54), who argued that physical activity is not limited to altering physical indicators but can also reshape daily time allocation and activity structure, thereby reducing time spent on smartphones. In other words, when girls become more physically active, a portion of their discretionary time may be reallocated from smartphone-based activities to offline pursuits, suggesting a possible daily time-allocation pattern in which higher physical activity co-occurs with lower smartphone use.

However, these findings should not be interpreted as evidence of a mediated effect whereby physical activity influences girls’ body shape perception through reduced smartphone use, because the present analyses did not formally test mediation. Therefore, the association between physical activity and smartphone use among girls should be interpreted as behavioral co-occurrence within individuals over time, not as evidence of a causal or mediated mechanism. Future studies using more intensive longitudinal designs, such as ecological momentary assessment or daily diary methods, may be better suited to examine whether changes in physical activity precede changes in smartphone use and whether such changes are subsequently linked to body-related perceptions.

In contrast, for boys, no statistically significant association emerged in this study, diverging from some prior findings suggesting that increases in physical activity are linked to reductions in problematic smartphone use (28, 30). This discrepancy may be related to the analytic strategy adopted here: by separating analyses by gender and applying a fixed-effects model to repeatedly observed panel data, the present study focused on within-person changes among the same boys over time rather than between-person differences across adolescents. In other words, any cross-sectional pattern in which boys who are generally more physically active also tend to use smartphones less would be largely absorbed by individual fixed effects. As a result, the remaining within-person association between changes in physical activity and smartphone use among boys may be small, unstable, or heterogeneous across individuals, leading to a non-significant estimate in the current models.

Although several coefficients reached statistical significance, their magnitudes were modest and should be interpreted in relation to both the measurement scales and the fixed-effects design. Subjective body shape perception was measured on a 5-point scale, and smartphone use was measured using 9-point categorical use-time scales rather than exact hours or minutes. Therefore, the observed coefficients indicate small within-person changes in scale scores, not large or immediately observable changes in body-related perceptions or smartphone-use duration. Accordingly, the findings should be interpreted as modest longitudinal associations rather than evidence of large practical effects.

These findings have practical implications for adolescent health promotion. For boys, because increases in physical activity were associated with lower subjective body shape perception scores, future studies may examine whether school and community-based activity programs such as physical education, organized sports, and other activity-based programs can support bodily competence, physical function, and positive engagement with the body. For girls, future intervention studies may consider combining physical activity promotion with smartphone use awareness and media literacy education, particularly to address appearance-related comparison in digital contexts. However, because the present study was observational and measured total smartphone use time rather than specific digital content, these implications should be interpreted as directions for future testing rather than direct evidence of intervention effects.

From a public health perspective, these patterns observed across the gender-stratified models suggest that population-level interventions (e.g., national school health policies) may need to move beyond one-size-fits-all approaches and consider tailored behavioral change strategies that recognize potentially different behavioral contexts among boys and girls. Such gender-responsive interventions can be incorporated into multi-layered school health strategies that integrate physical activity promotion, digital wellbeing curricula, and body image education to support healthier body-related perceptions during adolescence.

## 5 Conclusion

### 5.1 General conclusions

Using 5 waves of panel data and fixed-effects models, this study found different within-person association patterns in models estimated separately among boys and girls between adolescents’ behaviors and subjective body shape perception. Among boys, increases in physical activity were associated with lower scores on the subjective body shape perception scale, whereas among girls, increases in weekday smartphone use were associated with higher scores on the subjective body shape perception scale. Physical activity was also associated with lower smartphone use among girls. These findings suggest that physical activity, smartphone use, and subjective body shape perception may be connected in behavioral contexts observed separately among boys and girls. Future research and intervention studies may consider gender-sensitive approaches that emphasize physical activity opportunities for boys and integrate physical activity promotion, smartphone use awareness, and media literacy for girls.

### 5.2 Limitations and future research

Several limitations should be noted. First, the key variables were measured using single item indicators. Although the KYHPS provides valuable, repeated measures of adolescent health behaviors, brief survey items may not fully capture the multidimensional nature of physical activity, smartphone use, or body-related perceptions. Second, the dependent variable reflects subjective body shape perception rather than overall body image. Future research should therefore examine a broader range of body image dimensions, including affective and cognitive components such as body dissatisfaction, appearance anxiety, and appearance-based social comparison (1). Because this measure captures perceived body shape along a thinness–fatness continuum, lower scores should not automatically be interpreted as healthier body image, particularly among boys for whom muscularity, strength, and physical function may also be salient.

Third, the smartphone use measure captured total use time only and did not distinguish between different purposes or content types. Prior research has reported that females tend to use smartphones primarily for interpersonal communication (e.g., SNS and messaging), whereas males more often use them for entertainment purposes (e.g., games, videos, and music) (55–57). Therefore, smartphone activities may differ by gender and may not be equally relevant to body-related perceptions, and future studies should assess more detailed usage contexts, including appearance-focused social media exposure. Fourth, although this study examined the association between physical activity and smartphone use, it did not formally test mediation; therefore, the findings should be interpreted as behavioral co-occurrence rather than evidence of an indirect pathway. Finally, the analyses used a complete case approach and included adolescents observed at least twice across the five waves; thus, missing data and attrition may have influenced the analytic sample. In addition, although fixed-effects models reduce bias from stable time-invariant individual characteristics, they do not eliminate bias from time-varying confounding or reverse causation (58). Changes in mental health, peer relationships, sleep, dietary behaviors, or school context may have co-occurred with changes in physical activity, smartphone use, and subjective body shape perception. It is also possible that changes in subjective body shape perception influenced subsequent physical activity or smartphone use. Therefore, the findings should be interpreted as longitudinal within-person associations rather than evidence of direct causal effects.

Future longitudinal research would also benefit from incorporating more comprehensive behavioral measures, such as objective assessments of physical activity and more detailed indicators of smartphone use, to clarify the mechanisms linking daily behaviors to changes in adolescents’ body-related perceptions over time.

## Data Availability

Publicly available datasets were analyzed in this study. This data can be found at: https://www.kdca.go.kr/kdca/5462/subview.do.
